# Antifungal screening of selenium nanoparticles biosynthesized by microcystin-producing *Desmonostoc alborizicum*

**DOI:** 10.1186/s12896-023-00807-4

**Published:** 2023-09-27

**Authors:** Bahareh Nowruzi, Bilal Saad Jalil, James S Metcalf

**Affiliations:** 1 Department of Biotechnology, Faculty of Converging Sciences and Technologies, Science and Research Branch, Islamic Azad University, Tehran city, Iran; 2 Iraqi ministry of higher education and scientific research, Karbala University, Karbala city, Iraq; 3 Department of Biological Sciences, Bowling Green State University, Bowling Green city, OH 43403 USA; 4 Brain Chemistry Labs, Box 3464, Jackson, WY 83001 city USA

**Keywords:** Selenium nanoparticles, *Desmonostoc alborizicum* strain 1387, Toxic cyanobacterium, Antifungal screening

## Abstract

Metal nanoparticles exhibit excellent antifungal abilities and are seen as a good substitute for controlling different kinds of fungi. Of all known taxa, cyanobacteria have received significant consideration as nanobiofactories, as a result of the cellular assimilation of heavy metals from the environment. The cellular bioactive enzymes, polysaccharides and pigments can be used as reducers and coatings during biosynthesis. The probability of the antifungal activity of selenium nanoparticles (SeNPs) to prevent plant fungi that can affect humans was evaluated and a toxic Iranian cyanobacterial strain of *Desmonostoc alborizicum* was used to study the biotechnology of SeNP synthesis for the first time. Characterization of nanoparticles with a UV-Vis spectrophotometer showed the formation of SeNPs in the range of 271–275 nm with the appearance of an orange color. Morphological examination of nanoparticles with Transmission Electron Microscopy (TEM), revealed the spherical shape of nanoparticles. The results of X-Ray Diffraction (XRD) showed 7 peaks and a hexagonal structure of average crystal size equal to 58.8 nm. The dispersion index of SeNPs was reported as 0.635, which indicated the homogeneity of the nanoparticle droplet size. The zeta potential of the nanoparticles was + 22.7. Fourier-transform infrared spectroscopy (FTIR) analysis exhibited a sharp and intense peak located at the wave number of 404 cm^− 1^, related to the SeNPs synthesized in this research. The results of the antifungal activity of SeNPs showed among the investigated fungi, *Pythium ultimum* had the highest resistance to SeNPs (14.66 ± 0.52 µg/ml), while *Alternaria alternata* showed the highest sensitivity (9.66 ± 0.51 µg/ml) (p < 0.05). To the best of our knowledge this is the first report concerning the characterization and antifungal screening of SeNPs biosynthesized by Iranian cyanobacteria, which could be used as effective candidates in medical applications.

**Supplementary Information:**

The online version contains supplementary material available at 10.1186/s12896-023-00807-4.

## Introduction

Foodborne diseases have increased worldwide, with noticeable public health concerns. Furthermore, outbreaks and an increase in antibiotic-resistant microbes could endanger animal and human health resulting in a worldwide health crisis [1]. Additionally, a great diversity of stressors and free radicals may also affect the antioxidant defenses of biological systems [2]. Therefore, new approaches to dealing with these problems include natural products such as antimicrobial substitutes, which have antimicrobial activities and antioxidant effects [3], [1] [2], helping to alleviate oxidative stress through the use of improved non-enzymatic and enzymic antioxidants [4].

Given the harmful effects of agrochemicals such as fertilizers and pesticides on soils and aquatic environments, in recent years studies have focused on alternatives to these chemicals. Cyanobacteria and their extracts could be an attractive strategy within a sustainable agricultural framework [3, 4] as they may be a source of both biofertilizers [5, 6] and bioactive compounds for disease control in plants [7]. Biocontrol agents derived from cyanobacteria can modify the physiological and biochemical reactions of the host plant leading to the synthesis or supply of defense chemicals against pathogens such as antioxidants. Such changes can lead to increased plant growth, enhanced yields and quality of products. To our knowledge, the pioneering study of the use of cyanobacteria in the control of plant diseases was reported by de Caire et al. (1979), studying the effect of extracts from a nitrogen-fixing cyanobacterium, *N. muscorum* (Nostocaceae), on the growth of millet (*Panicum miliaceum* L. (Poaceae)) seedlings. Later, the cyanobacterial biological control mechanism against plant diseases was studied in tomato (Solanaceae) plants infected with *Fusarium* (Nectriaceae) wilt and which were treated with two cyanobacterial species, *Anabaena variabilis* and *A. laxa* (Nostocaceae) [8]. In recent years, there has been a growing worldwide interest in the use of cyanobacterial biomass as biofertilizers to replace chemical fertilizers, in part to overcome increasing organic-farming demands. In this regard the antifungal potential of extracellular cyanobacterial media from *Anabaena subcylindrica*, *Nostoc muscorum* (Nostocaceae) and *Oscillatoria angusta* (Oscillatoriaceae) was evaluated on various pathogenic fungi isolated from different organs (leaves, stems and roots) of the faba bean (Fabaceae) and significantly inhibited fungal growth isolated from the three plant parts [9]. Moreover, cyanobacterial extracts have been reported to reduce the occurrence of *Botrytis cinerea* (Sclerotiniaceae***)*** on strawberries and *Erysiphe polygoni* (Erysiphaceae), responsible for powdery mildew on turnips and damping-off in tomato seedlings (Solanaceae), as well as reducing saprophytes such as *Chaetomium globosum* (Chaetomiaceae), *Cunninghamella blakesleeana* (Cunninghamellaceae) and *Aspergillus oryzae* (Trichocomaceae) and plant pathogens such as *Rhizoctonia solani* (Ceratobasidiaceae) and *Sclerotiana sclerotium* (Sclerotiniaceae) [10]. Methanolic extracts of *Nostoc commune* FK-103 (Nostocaceae) and *Oscillatoria tenuis* FK-109 (Oscillatoriaceae) showed antifungal activities against *Phytophthora capsici* (Pythiaceae), an oomycete plant pathogen that causes blight and fruit rot of peppers and other important commercial crops. A methanolic extract of *Nostoc commune* FK-103 (Nostocaceae) inhibited the growth of *Rhizopus stolonifer* (Mucoraceae), a fungus responsible for soft and liquid rots on various fruits and vegetables (strawberries (Rosaceae**)**, grapes (Vitaceae), tomatoes (Solanaceae)) as well as black mold, commonly observed on the surface of bread and other starchy foods [11]. Manjunath et al. (2010) investigated the biocidal effectiveness of *Calothrix elenkenii* (Rivulariaceae) fungicidal compounds toward damping-off infection in three vegetable crops-tomato, chili, and brinjal (Solanaceae), and results revealed that the *Calothrix elenkenii* (Rivulariaceae) ethyl acetate extract was successful against the fungi examined [12]. Chaudhary et al. (2012) reported that two-cyanobacterial species *Anabaena variabilis* and *A*. *oscillarioides* (Nostocaceae) prevented damping off infection in tomato seedlings (Solanaceae) threatened by a fungal consortium including *Pythium debaryanum* (Pythiaceae), *Fusarium oxysporum lycopersici, Fusarium moniliforme* (Nectriaceae), and *Rhizoctonia solani* (Ceratobasidiaceae). Moreover, Roberti et al. (2016) reported that an extract of *Anabaena* sp. (Nostocaceae) showed considerable inhibitory activity against the plant pathogen *Podosphaera xanthii* (Erysiphaceae*)*, responsible for powdery mildew on cucurbits such as zucchini (*Cucurbita pepo* (Cucurbitaceae)) cotyledons [13]. The use of extracellular media from *Oscillatoria* sp. (Oscillatoriaceae) and *Nostoc muscorum* (Nostocaceae) can regulate the purple blotch disease of onion induced by *Alternaria porri* (Pleosporaceae) [14]. As part of the biological control of *Fusarium oxysporum* f. sp. lycopersici (FOL) (Nectriaceae), a xylem-colonizing fungus that has long been known to cause wilt on tomato (Solanaceae), Alwathnani and Perveen (2012) reported that the methanolic extract of two cyanobacterial species *Nostoc linckia* (Nostocaceae) and *Phormidium autumnale* (Oscillatoriaceae) exhibited moderate inhibitory activity against the growth of this fungus [15]. However, Kim and Kim (2008) showed that extracts of the cyanobacterium *Nostoc commune* FK-103 (Nostocaceae) inhibited in vitro and in vivo growth and sporulation of the tomato-wilt (Solanaceae) pathogen in tomato (Solanaceae) seeds [16]. Moreover, the antifungal potential of cyanobacteria on the tomato-wilt (Solanaceae) pathogen was also evaluated with extracts of two other species of cyanobacteria, *Anabaena variabilis* and *A. laxa* (Nostocaceae). The results showed that this new type of biological control was effective and allowed both reduction of disease intensity alongside increased growth/production of tomato (Solanaceae) crops affected by *Fusarium* wilt (Nectriaceae) [17].

In fact, biocontrol agents derived from cyanobacteria can modify the physiological and biochemical reactions of the host plant leading to the synthesis or supply of defense chemicals against pathogens such as antioxidants. Such changes can lead to increased plant growth, enhanced yields and quality of products.

The use of nanoparticles against various pathogens is now well recognized in the agriculture and health sectors. Nanoparticles have been shown to exhibit various novel properties and these properties rely upon the size, shape, and morphology of these particles. Moreover, these physical characteristics enable them to interact with microbes, plants, and animals. Smaller-sized particles have shown more toxicity than larger-sized nanoparticles [18–27]. Consequently, we decided to use an Iranian cyanobacterial strain to biosynthesize nanoparticles and evaluate their antifungal effect.

Selenium (Se) is a crucial micronutrient that has received noticeable attention due to its vital functions in biological systems [5]. This element is the crucial constituent of selenoprotein which is famously known to participate in the animal cells’ anti-oxidant defense system [6]. Furthermore, it has been shown that biogenic selenium nanoparticles possess very good antimicrobial activity against clinical isolates of *P. aeruginosa* but lower efficacy toward *C. albicans* [7]. Moreover, the antifungal activity of selenium nanoparticles synthesized by *Bacillus* species Msh-1 towards *Aspergillus fumigatus* and *Candida albicans* was investigated by Shakibaie et al., [28].

Nanotech is an emerging interdisciplinary technique covering many areas of theoretical investigation [29, 30] [8]. It is capable of assisting revolutionary applications in animal and human health, including resistance to e.g. pathogens, antioxidants, breakdown of toxins, nutrient performance [9]. Due to the fact that Se in nano form is thought to be more efficient and have less costly anti-microbial and anti-oxidant activities, it has obtained a great deal of attention and broad application over the past few years [31], [32–34].

Bionanoparticles of Se may be manufactured using bacteria as biocatalysts, developing a secure and environmentally responsible innovation strategy to manufacture metal/metalloid nanoparticles with low toxicity and high bioactivity without the necessity to stabilize or reduce the agents [11], [35], [36]. The nutritional and medicinal properties of cyanobacteria were evaluated for the first time in 1500 BC, with *Nostoc* species used treat fistulas, gout and some types of cancer [12].

*Desmonostoc* species are an important source of bioactive compounds and belong to the Nostocaceae family, which contains a variety of bioactive substances such as carotenoids, triterpenoids, amino acids, phenolics, sulphates polysaccharides, phycocyanin and poly-unsaturated fatty acids and these components may have bactericidal, antioxidant and antimicrobial activity [13]. A microcystin-producing strain of *Desmonostoc alborizicum* was isolated from a water source system in Iran [34] and this strain could also have useful antifungal properties.

In this study, we explored the biosynthesis of SeNPs via a single-step reduction of sodium selenite solution using a cyanobacterial strain at room temperature without the aid of any reducing and/or capping agents. To our knowledge, this is the first scientific report on the synthesis of SeNPs from cyanobacteria in a non-hazardous way and the monitoring of their antifungal activity against pathogenic plant fungae.

## Materials

### Cultivation and preparation of the extracts

The cyanobacterial strain was obtained from the Cyanobacteria Culture Collection (CCC) affiliated with the Science and Research Branch of the Islamic Azad University, Tehran, Iran. At the lab, little fragments of the cyanobacterial mat were spread into 1.2%-agar-solidified BG-11_0_ medium and cultivated. There was constant microscopic analysis of biomass and consecutive striations were carried out until a monocyanobacterial colony was obtained [37].

The isolate was given a temporary appointment of 1387 and kept in a 250 mL cotton-stoppered Erlenmeyer flask including liquid BG-110 medium at 28 ± 2 ºC with regular trembling (twice a day), illumination of ca. 50–55 µmoL photons m^− 2^s^− 1^, and a regime of 14:10 h light: dark cycle [14].

After 15 days, the cyanobacterial extract was dried and powdered. In general, 5 g (dry weight) of biomass was suspended within 100ml of double sterile distilled water for 15 min at 100 °C in an Erlenmeyer flask. After boiling, the mixture was cooled and centrifuged at 10,000 rpm for 15 min. The supernatant was collected and was stored at 4 ºC until further analysis. The cell free extracts of the cyanobacterial strain for NPs synthesis were used within 1 week of preparation [38].

### Preparation of SeNPs

Biological synthesis of SeNPs is done using the following procedures; In the typically synthesis process of SeNPs, 2 ml of pure microalgal extract was drop wise added into 100ml of 5 mM sodium selenite solution. The reaction mixture was kept at 60 °C under mechanical stirring as the colourless solution gradually became red to orange after constant stirring for 10 min [39].

### Characterization of SeNPs

#### UV-Visible spectroscopy

Formation of SeNPs was analyzed by UV-Vis spectrometry in the wavelength range of 200–800 nm. Sampling was performed with one ml of solution. The synthesized SeNPs underwent centrifugation at 10,000 rpm for 10 min. The pellet was suspended in deionized water, centrifuged again, and lyophilized. The lyophilized SeNPs (1.31 mg) were dissolved in distilled water and used for different characterization studies [40].

#### Transmission electron microscope (TEM) analysis

The shape and size of SeNPs were measured by TEM (JEOL 1010, Japan). Samples were prepared by placing 5ml of SeNPs suspension on a carbon-coated copper grid. Grids were dried under an infrared lamp and inspected with TEM. Samples were observed by using TEM (CM-200, Phillips) operated at an accelerating voltage of 200 kV. Average size of SeNPs was determined by measuring at least 100 particles [41].

### FTIR analysis

The Infrared spectrum of nanoparticles gives information about the functional groups or the nature of that particular compound. IR spectra of NPs was carried out on a Shimadzu-8400 S FTIR spectrophotometer (Shimadzu, Japan) using the spectral region of 400-4000 cm^− 1^ with a resolution of 4 cm^− 1^ and 64 co-added scans. All the colloidal nanoparticles were dried in the form of nano-powders and palletized with KBr for FTIR studies [42].

### X-ray diffraction

Powder X-ray diffraction (XRD) was used to identify the crystalline nature of SeNPs with a detector voltage, Cu/Kα radiation and a current of 40 mA at a range of 20°–80° at 2θ. Size calculation was measured using Debye Scherrer’s Eq. [43].

D = Kλ / βCosθ.

where, K is a constant (with hypothetic value ≈ 0.9) λ indicates the wavelength of X-ray radiation, D denotes crystal size, θ represent Bragg’s angle and β shows full width at half maximum of the diffraction peak [44].

### Dynamic light scattering (DLS)

The size and size distribution of particles in the colloids were measured using a Nano ZS zetasizer system (Malvern Instruments). Measurement parameters were as follows: a laser wavelength of 633 nm (He–Ne), a fixed scattering angle of 173°, a measurement temperature of 25 °C, a medium viscosity of 0.8872 mPa⋅s, a medium refractive index of 1.330, and a material refractive index of 1.59. Before DLS measurement, the colloid was passed through a 0.2 μm polyvinylidene fluoride (PVDF) membrane. The sample was loaded into a quartz microcuvette [45].

### Zeta potential analysis

Zeta potential of the biosynthesized SeNPs aqueous suspensions was determined by placing 2 ml of the samples in a four-sided clear plastic cuvette in a Malvern zeta sizer instrument (Malvern instrumentation Co.) at 25 °C. The measurement was performed directly in the clear disposable zeta cell [46].

### Antifungal activity of nanoparticles

Various concentrations of 5 and 10 µg/ml SeNPs were tested for their antifungal activity using the paper disk diffusion method. Fungal isolates (*Botrytis cinerea, Alternaria alternate, Colletotrichum gloeosporioides, Fusarium oxysporum, Phytophthora capsici, Pythium ultimum, Rhizopus stolonifer*) were obtained from the Iranian Research Organization for Science and Technology (IROST) and stored at 4 °C on Sabouraud dextrose agar (SDA) slants. A loop of tested fungi slant was mixed in 5 mL of SDA and incubated at 28 °C until a turbidity value between 0.09 and 0.11 at 530 nm was reached. Absorbance values were measured using a microplate spectrophotometer (Infinite M200, Tecan) [47]. Different concentrations of SeNPs (5 and 10 µg/ml) were dissolved in 1 mL of 5% (v/v) DMSO. Sterilized paper discs (6 mm) were prepared. The previously saturated discs (6 mm) were placed on sides of SDA plates. SDA plates were incubated at 28 °C for five days. The inhibition zones (mm) around discs were measured by a transparent ruler. The antifungal agent Nystatin (10 µg) was included in the assays as a positive standard antibiotic control.

### Statistical analysis

Statistical assessment of the findings was produced with SPSS 16.0 (SPSS Inc. Chicago, Illinois, USA). All values were reported as an average ± SEM. The data were statistically analyzed by one-way analysis of variance (ANOVA). Given the exploratory nature of the study, likelihood values of P < 0.05 were considered statistically significant.

## Results

### Visual observation and optical analysis by UV-Vis spectrophotometer

**Fig. 1:**
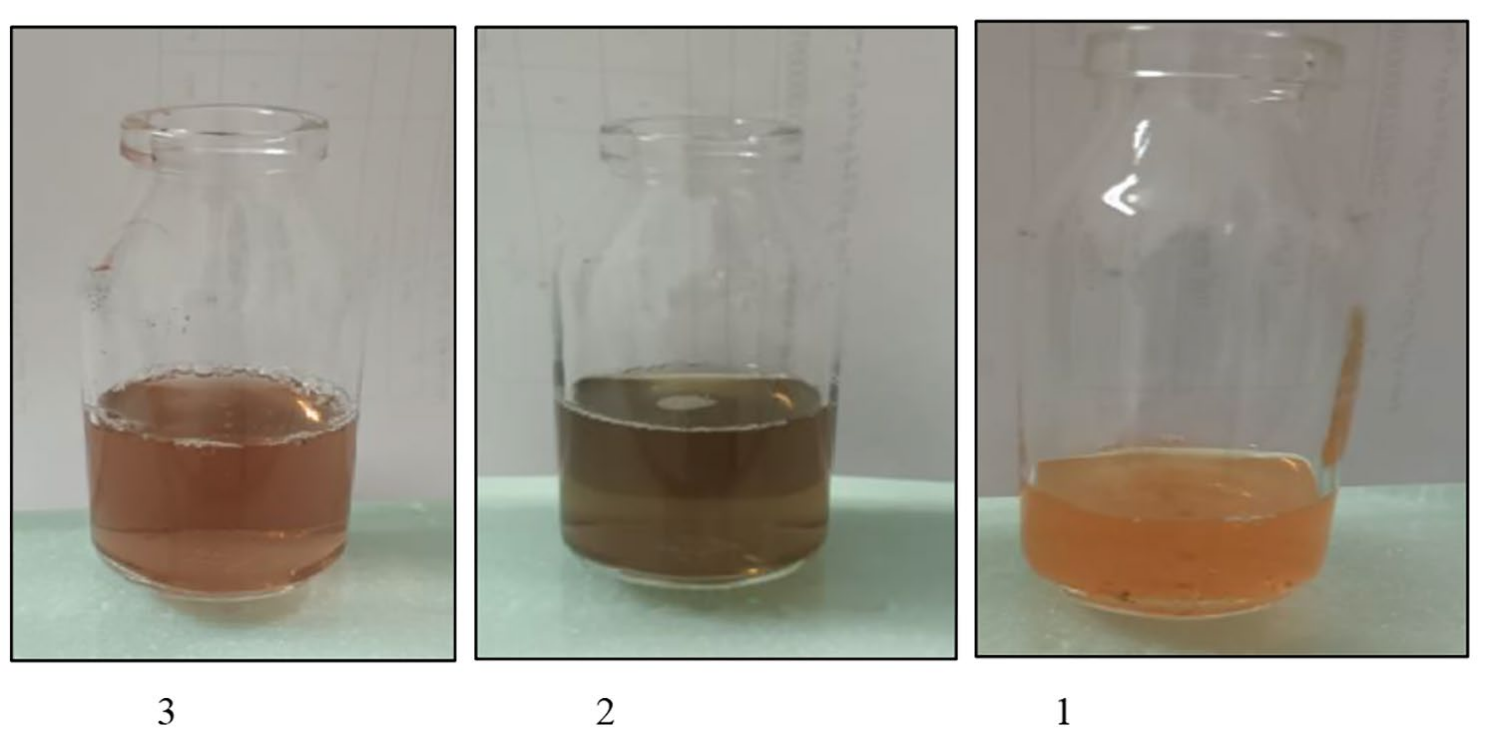
Reduction of sodium selenite to SeNPs by an increase in time. 1: zero time, 2: 12 h, 3: 24 h

**Fig. 2:**
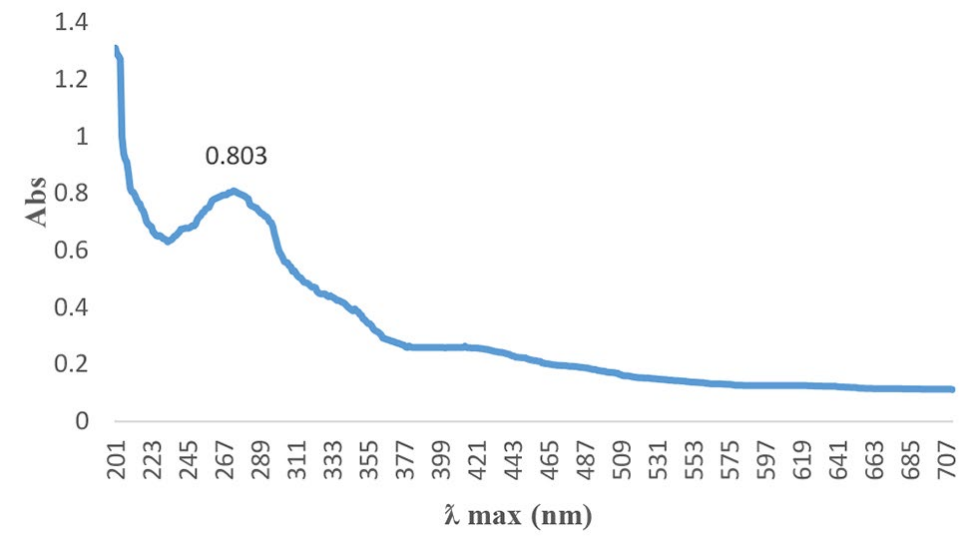
UV–Vis spectra of SeNPs

With time, the colour intensity of the SeNPs increased, indicating a gain in concentration. Furthermore, the reduction of sodium selenite solution by the reducing agent in the cyanobacterial extract was indicated by an initial light yellow to final red-orange color change (Fig. 1), which was further confirmed by UV-Vis spectrophotometry analysis, showing the UV-Vis spectrum of the SeNPs (Fig. 2). The maximum UV–visible absorption of SeNPs was found to be between 271 and 275 nm, due to plasma resonance of the SeNPs.

### TEM analysis

**Fig. 3:**
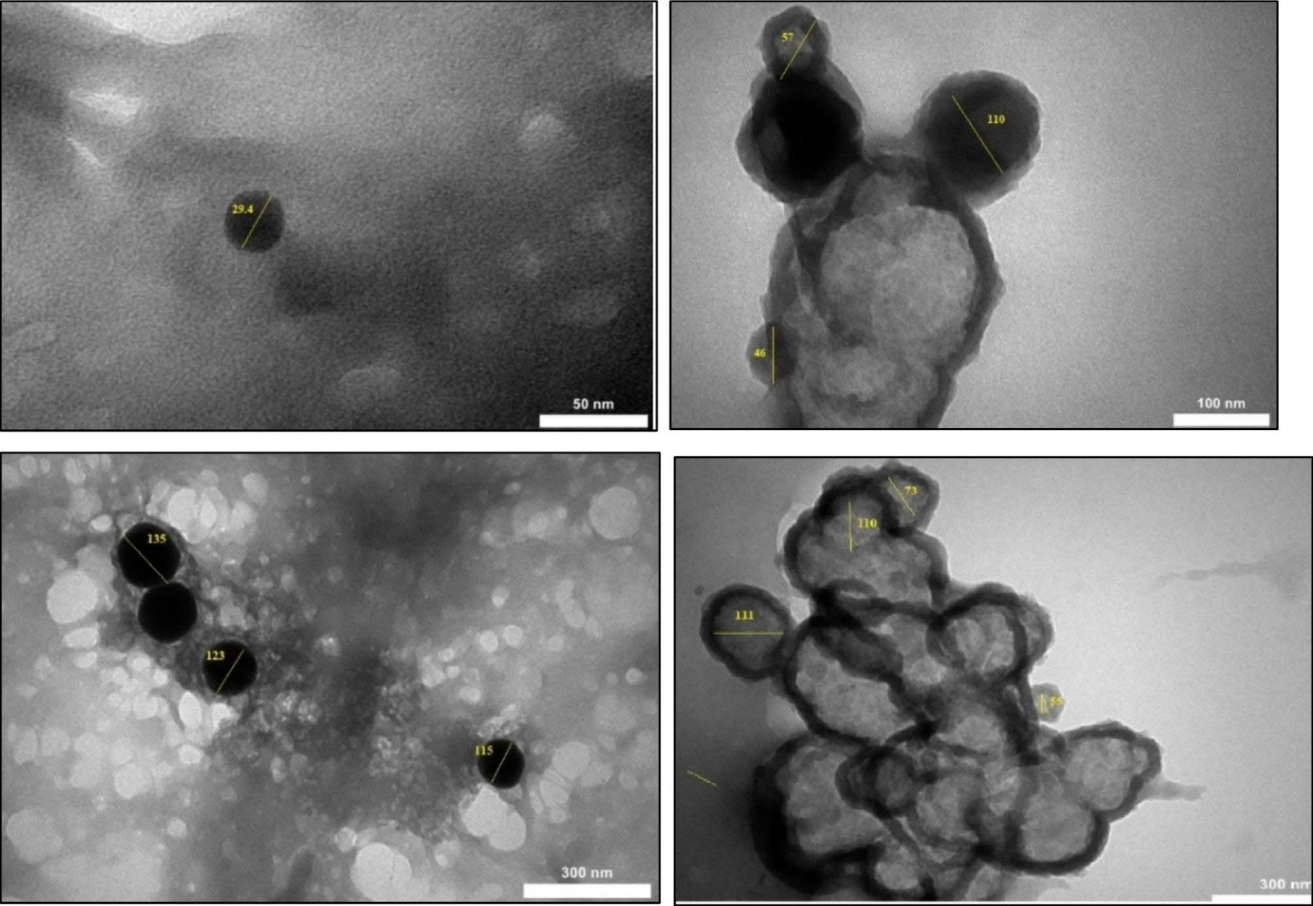
TEM images of biogenic SeNPs at different magnifications (50, 100 and 300 nm)

At 50 nm magnification, a single SeNP with a diameter of 29.4 nm was observed, while at the magnification of 100 nm, the state of agglomeration of nanoparticles and their accumulation was observed, showing a size of nanoparticles between 46 and 110 nm. This single and agglomerated state shows the random dispersion of SeNPs in the solution. The dispersion of nanoparticles was also confirmed at 300 nm magnification, with single and spherical nanoparticles at a size of 115 to 135 nm observed. Furthermore, at 300 nm magnification, the accumulation and agglomeration of SeNPs with a size between 55 and 111 nm was observed. By carefully observing the accumulation of nanoparticles, it is possible to distinguish between spherical and side-by-side nanoparticles. This accumulation and agglomeration can be attributed to sublimation and evaporation during the extraction and synthesis of SeNPs. The production process, the adhesive properties of stabilizers, the destruction of the surface coating of nanoparticles led to significant changes in the morphology of biomolecules, which further affects the aggregation. The final morphology strongly depends on the choice of stabilizers. Spherical particles are the most abundant because this morphology provides a lower level of surface energy (Fig. 3).

### FT-IR analysis

**Fig. 4:**
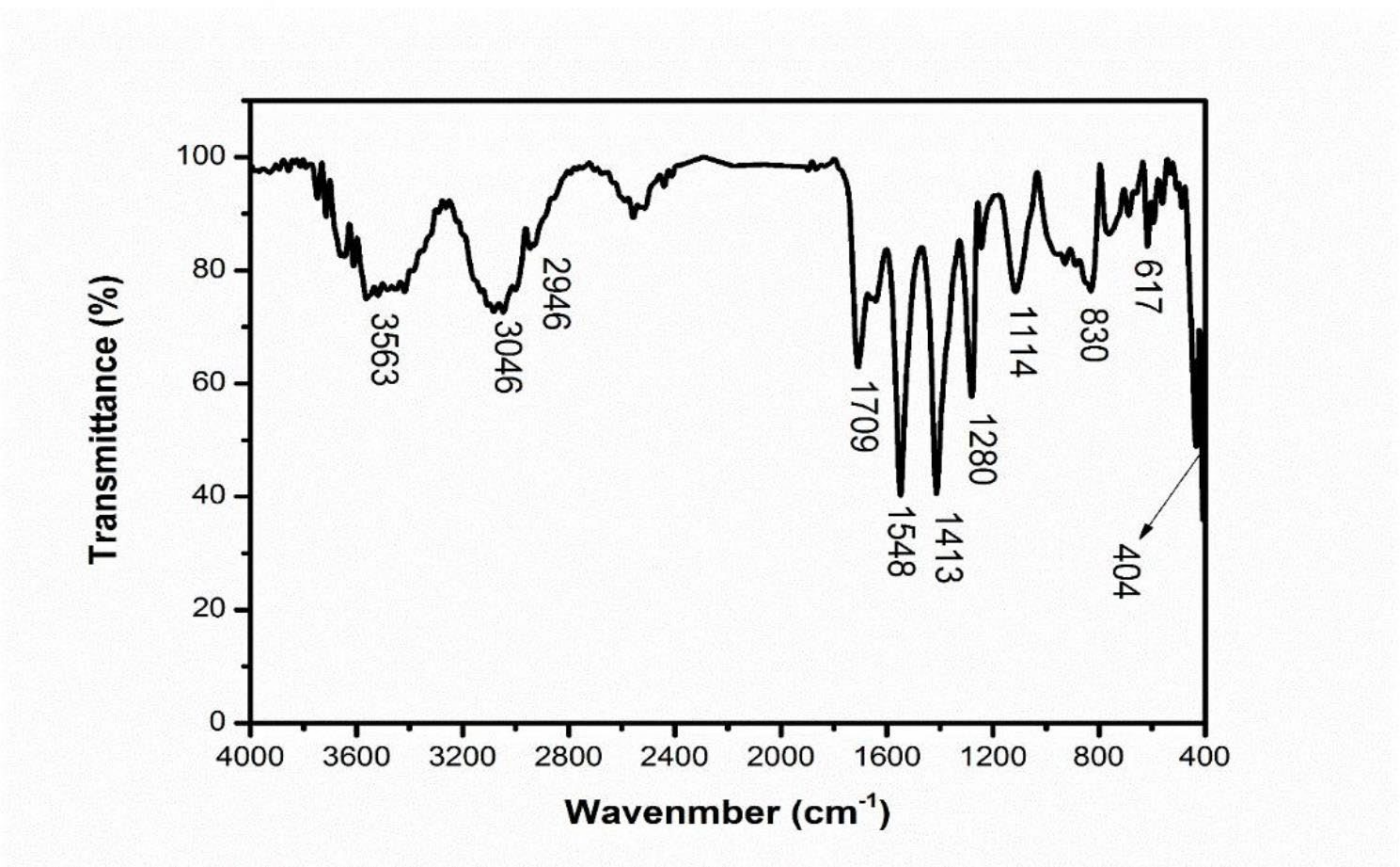
FT-IR spectrum of SeNPs synthesized

The obtained FTIR spectroscopy results from the SeNPs are shown in Fig. 4 and Fig. S1. The broad peak in the range of 3611 and 3644 cm^− 1^ in the first method is related to the presence of water molecules, which indicates O-H and N-H stretching vibrations and the presence of structural moisture caused by water vapor or O-H in alcohols and Phenols in the structure (Table 1). The shoulder peak in the range of 3046 − 2946 cm^− 1^ indicates OH bonds in carboxylic acids. Also, in this area, there are C-H bonds related to methyl groups (CH_3_), which overlap with the previous bonds. Peaks related to amine groups and N-H bond, which are amide bonds (amide I, II and III) are visible at wave numbers of 1709, 1548 and 1413 cm^− 1^, respectively. There are also C = O groups related to the ester bond in wave number 1709 cm^− 1^, which overlaps with amide I. The presence of the peak at 1280 cm^− 1^ indicates the stretching vibrations of aromatic amine bonds; Also, in this range, there are expected O-P = O bonds that overlap with the amide. The peak at 1114 cm^− 1^ is related to the stretching vibrations of the C-N bond in alcohol, ester, carboxylic acid and ether groups. The peak in the range of 830 cm^− 1^ corresponds to C-C and C-O bonds in the protein chain. The peak at 617 cm^− 1^ is also related to the C-O primary alcohol, C-O stretching vibrations and disulfide (C-S) knots of the bacterial protein chain (Table 1). In this region, there is also the presence of stretching vibrations of phosphoryl groups. The sharp and intense peak located at the wave number 404 cm^− 1^ is related to the SeNPs synthesized in this research. These findings verify the presence of functional biomolecules (phenols, polysaccharides and protein) mounted on the surface of the SeNPs .

**Table 1:** functional groups identified in different wave numbers of synthesized SeNPs.

Wavenumber (cm^− 1^)	functional groups	Peaks corresponding to functional groups
3563	The presence of structural moisture caused by water vapour O-H present in alcohols and phenols present in the structure of cyanobacterial extract	O-H vibrational bond
3563		N-H bond
2946–3046	OH bonds in carboxylic acids	Shoulder spade
2946–3046	CH_3_	C-H bonds
1413, 1548, 1709	Amine groups and N-H bonds that are amide bonds	Amide peaks of I, II and III
1709	Ester linkage	C = O bond
1280	Aromatic amines	Bonding stretching vibrations of amine
1114	Alcohol, ester, carboxylic acid and ether groups	C-O bond stretching vibrations
830	Bending vibration of aromatic ring of cyanobacterial extract	Bonding C-H stretching vibrations
617	Primary alcohol	C-O bond stretching vibrations
404	Synthesized SeNPs	Sharp and intense peak

### XRD analysis

**Fig. 5:**
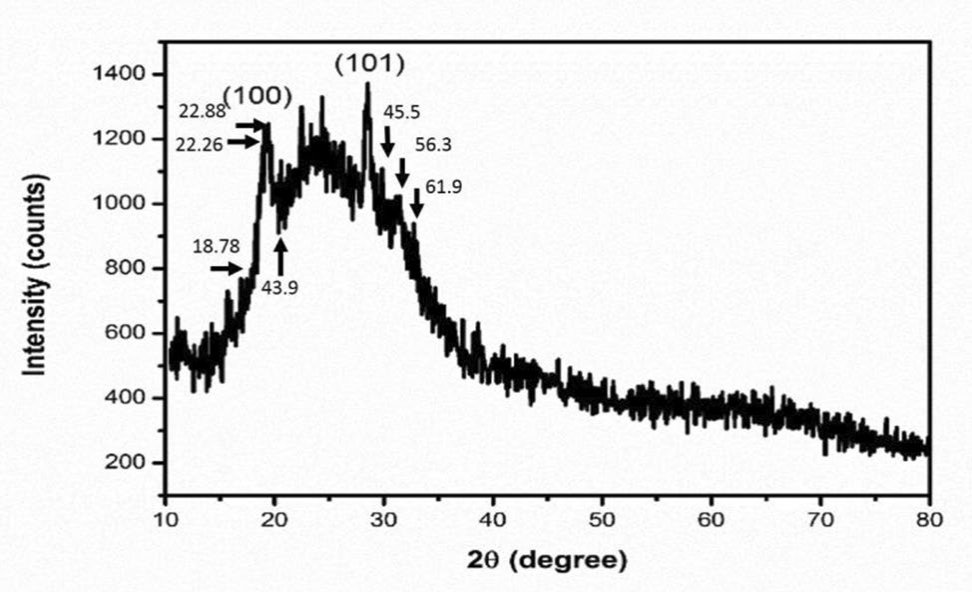
X-ray diffraction patterns related to assessment of SeNPa

The obtained XRD spectroscopy results are shown in Fig. 5 and Fig. S2. According to Fig. 5, it is clear that the synthesized sample has a semi-crystalline structure, which is caused by the presence of significant amounts of organic compounds obtained from the cyanobacterial extract on the surface of the synthesized Se crystalline nanoparticles, and in other studies, such a diffraction pattern is also observed by the green method.

The presence of a peak at 22.88 indicates the release of polysaccharides from the cyanobacterial extract. The sharp peak at 18.78 is related to the release of polysaccharides, which can overlap with the amorphous phase. The peak in the range of 22.26 indicates the crystallization of the inorganic phase. The peaks at 43.9 (102), 45.5 (111), 56.3 (112) and 61.9 (202) are related to the hexagonal structure of Se. In this sample, only one crystalline phase has been identified, and that is the Se structure (reference code JCPDS No. 00-006-0362) with a trigonal crystal structure. In this diffraction pattern, the diffraction planes (100) and (101) are placed at angles of 20.4° and 30.1°, respectively. The lattice constants in this sample are equal to a = 4.37 A° and c = 4.96 A°, which are close to the values reported in other studies. Scherer’s relation (relation [1]) is used to find the size of the crystal.

 [1] D = Kλ/(FWHM)×cos(θ).

In this relation, D is the size of the crystal / K is the shape factor / λ is the wavelength of the X-ray used (1.54 Angstroms), FWHM is the bandwidth at half height and θ is the location of the peak. Having the value of cos (θ) and FWHM, as well as the fixed values of λ (1.54 angstroms) and k (0.9), the value of the crystal size is obtained according to Scherer’s relation, which considering that λ was in angstroms, this value is also in angstroms. By dividing the obtained value by 10, the crystal size can be calculated in nanometers. This parameter for the Se phase in the examined sample was equal to 58.80 nm (Fig. 5).

### DLS results

**Fig. 6:**
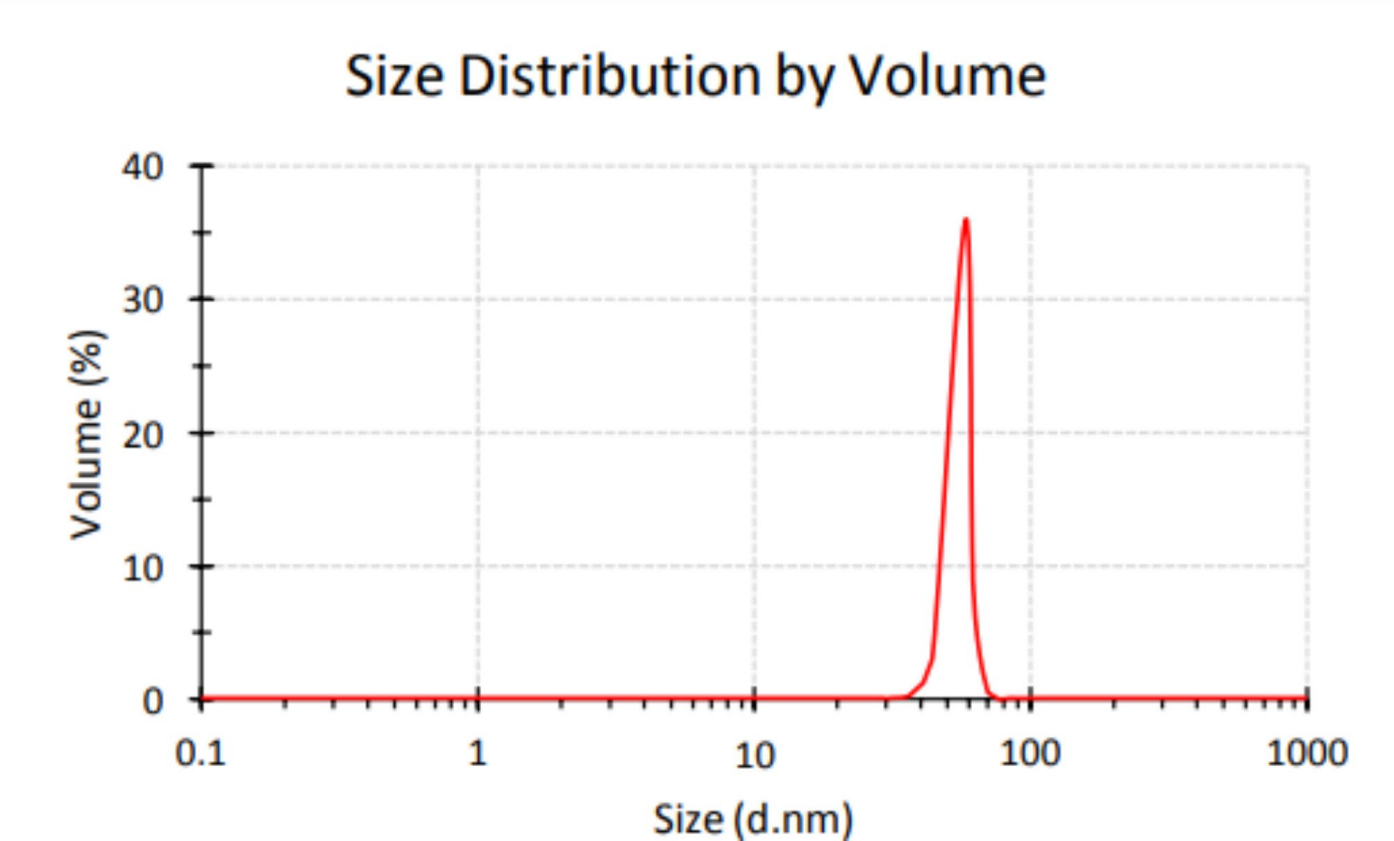
Size distribution of synthesized SeNPs.

The size of the particles in nanometers, their amount in percent, as well as the selected peaks of SeNPs are presented in Fig. 6. The obtained curve was a single peak with a width of 29 nm. The results of this research showed that the frequency of particle size was 0.2% (32.67 nm), 1.2% (37.84 nm), 3.2% (43.82 nm), 8.2% (50.75 nm), 84.5% (58.77 nm), 2.2% (68.06 nm) and 0.5% (78.82 nm). The obtained results were completely consistent with the results of the dimensions of nanoparticles obtained from the XRD results. The value of polydispersity index (PDI) was reported as 0.635. PDI indicates the homogeneity of droplet size in nanoparticles (fig. 6) (Table 2).

**Table 2:** Results of size distribution of SeNPs.

(%)	Size of particle (nm)	Peak
0.2	32.67	Peak 1
3.2	37.84	Peak 2
3.2	43.82	Peak 3
8.2	50.57	Peak 4
84.5	58.77	Peak 5
2.2	68.06	Peak 6
0.5	78.82	Peak 7

### Zeta potential analysis

**Fig. 7:**
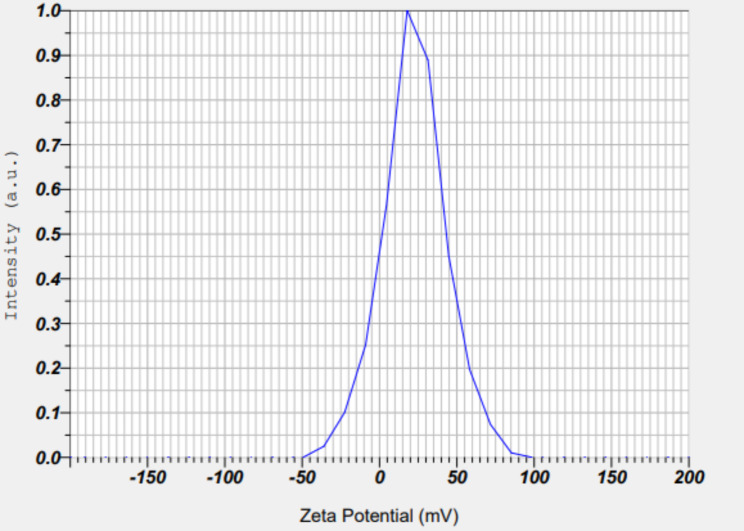
Zeta potential of SeNPs

The results of the zeta potential diagram of the produced nanoparticles (Fig. 7) showed that the zeta potential of the produced SeNPs was equal to 22.7 mV and the particles had an electrophoretic mobility of 0.117 Vs/cm^2^, showing the stability of synthesized SeNPs. This result is in good agreement with the results obtained from DLS measurements. The stability of nanoparticle systems is important from the aspect of application, especially the interaction with biological organisms. Unstable systems are not reliable because their interaction can be easily changed by the accumulation of particles (Fig. 7).

### Antifungal activity of SeNPs

Table 3 show the average results of the antifungal activity of SeNPs synthesized with *Desmonostoc alborizicum* cyanobacterial extract at two concentrations of 5 and 10 µg/ml. Nystatin was used as a positive control in this study. According to the presented results, the antifungal activity of SeNPs synthesized in different concentrations showed a significant difference with respect to each other (p < 0.05). Examining the results of antifungal activity of SeNPs showed that at a concentration of 1 µg/ml, inhibitory effects occurred with tested fungal strains. By increasing the concentration of SeNPs from 5 µg/ml to 10 µg/ml, an increase in fungicidal properties and in fact an increase in the diameter of the growth halo of the investigated fungi was observed. *Alternaria alternata* was the most sensitive among the studied fungi, followed by *Phytophthora capsid* and *Botrytis cinerea* (p < 0.05). The most resistant fungus against SeNPs was reported in *Pythium ultimum*, and the fungi *Colletotrichum gloeosporioides, Fusarium oxysporum* and *Rhizopus stolonifer* showed the highest resistance against SeNPs synthesized from the extract of cyanobacterium *Desmonostoc alborizicum* (p < 0.05).

**Fig. 8:**
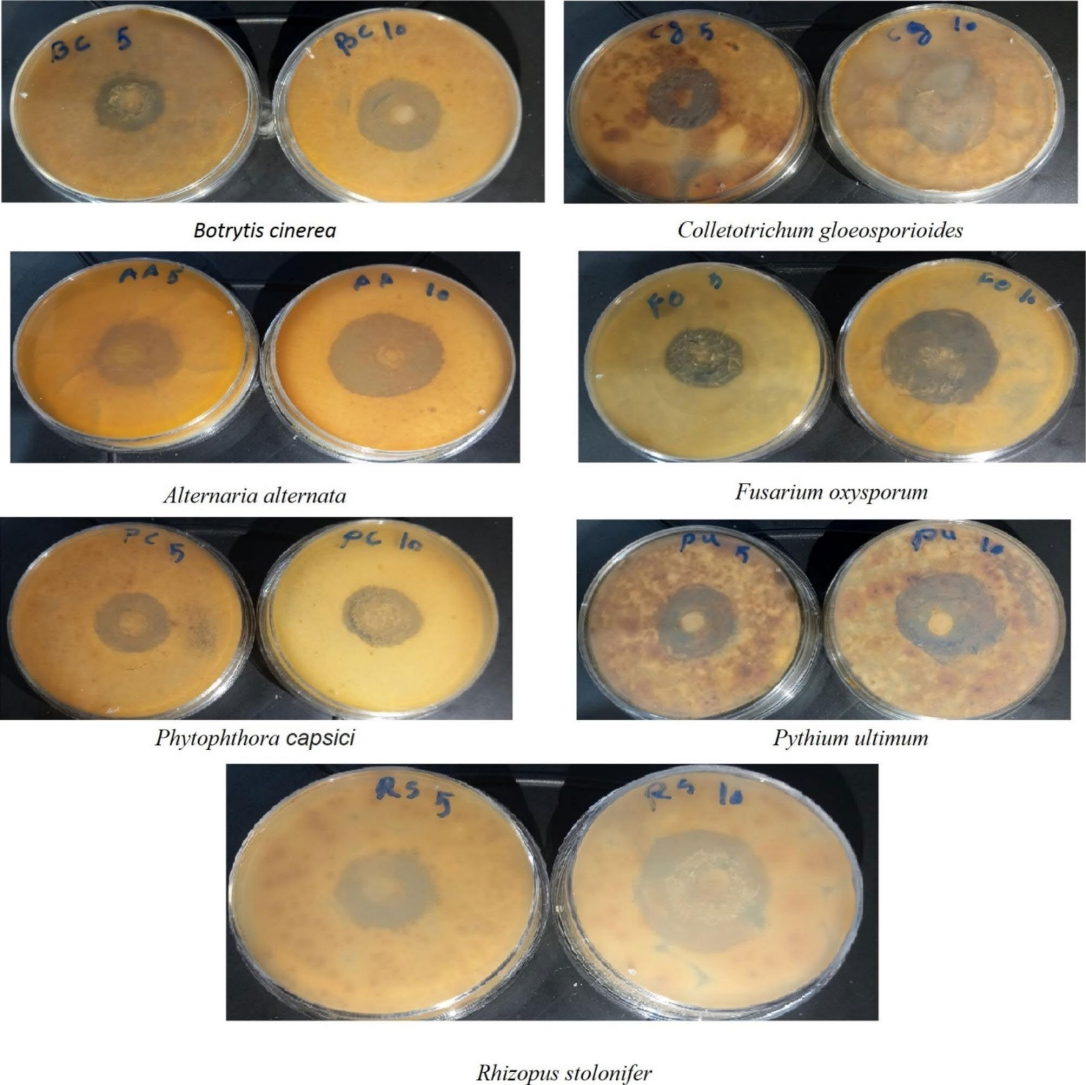
Antifungal activity of SeNPs against plant pathogenic fungae

In general, using a concentration of 10 µg/ml significantly increased the diameter of the lack of growth halo (increased inhibition of growth) of the investigated fungi (p < 0.05). Among the investigated fungi, *Pythium ultimum* showed the highest resistance to SeNPs (p < 0.05), while *Alternaria alternata* showed the highest sensitivity (p < 0.05) (Fig. 8)(Table 3) (fig. S3).

**Table 3:** Comparison of non-growth diameter of SeNPs against plant pathogenic fungae. Different letters indicate significant differences (p ≤ 0.05)

fungus	non-growth diameter (mm)		
	Nystatin (10 µg/ml)	10 µg/ml of SeNPs	5 µg/ml of SeNPs
*Botrytis cinerea*	19.66 ± 0.07 a	12.66 ± 0.57 c	9.66 ± 0. 7 bc
*Alternaria alternata*	18.33 ± 0.57 a	9.66 ± 0.51 d	7.66 ± 0.6 d
*Colletotrichum gloeosporioides*	19.33 ± 0.52 a	14.33 ± 0. 7 ab	10.66 ab
*Fusarium oxysporum*	18.66 ± 0.59 a	13.33 ± 0.6 bc	10.33 ± 0.52 ab
*Phytophthora capsici*	18.33 ± 0.6 a	10.66 ± 0.51 d	8.66 ± 0.52 cd
*Pythium ultimum*	20 ± 1 a	14.66 ± 0.52 a	11.33 ± 0.51 a
*Rhizopus stolonifer*	16.66 ± 0.6 a	13.66 ± 0.56 abc	10.66 ± 0.56 ab

## Discussion

Different species from the family Nostocaceae can inhibit the increase of several microorganisms because they are high in bioactive ingredients with antimicrobial activity. A previous study [37] showed that a new species of *Desmonostoc alborizicum* can produce microcystins. The massive presence of cyanotoxins is not just a water quality issue, but also a grave danger for animal and human health. Such toxins include powerful hepato- and neurotoxic, as well as dermato- and cytotoxic agents manufactured by multiple cyanobacterial genera and species [14]. Several cases of lethal poisoning in animals due to these toxins have been reported worldwide in recent decades [31].

In general, within freshwaters, blooms containing hepatotoxic agents are more frequent than those containing neurotoxins [47]. In the present study the antifungal activity of SeNPs biosynthesized by a *Desmonostoc alborizicum* microcystin-producing strain 1387 was evaluated and results revealed that SeNPs synthesized by this strain exhibited antifungal activity against plant pathogenic fungae.

Selenium acts as a vital trace component for every living organism and is an essential nutritional ingredient incorporated into various proteins that help the body’s immune system work appropriately, stop cell damage, and adjust thyroid gland function, along with having strong anti-cancer and bactericidal effects. Selenium is also a cofactor in various enzymes such as thioredoxin reductases and glutathione peroxidases. Selenium as selenite and selenate is toxic compared to SeNPs, which are not toxic [48].

Microbial synthesis of SeNPs is a two-step reduction process from SeO_4_(^2−^) to SeO_3_(^2−^) and then to insoluble elemental selenium (Se0). These reactions are catalyzed by selenate and selenite reductases [17]. However according to the studies of [33], the secondary metabolites found in cyanobacteria are responsible for the synthesis of SeNPs, while [34] suggested a reductase enzyme present in the cyanobacterial extract as the agent of synthesis.

Various studies have been presented concerning the synthesis of nanoparticles from cyanobacteria. Alipour et al. [49] reported 450 to 500 nm nanoparticles produced from *Spirulina platensis* during UV-visible spectroscopy. Afzal et al. [40] investigated the synthesis of SeNPs from an extract of the cyanobacterium *Anabaena variabilis*, reporting a peak in the UV spectrum at 266 nm. They also stated that the maximum absorption of the extract at a wavelength of 280 nm may be due to the presence of protein in the extract and at 614 nm due to the presence of phycobilin pigments. Also, Srivastava et al. [50] reported a λmax of SeNPs synthesized from *Zooglea ramera* at 330 nm. The difference in the UV spectrum produced by SeNPs using bacterial reduction has been reported to be due to the difference in their atomic structure and size [51]. In addition, color changes from orange to red during the formation of SeNPs have been reported for the fungi *Gliocladium roseum* and *Mariannaea* sp. HJ [52] [53] and by the bacterium *Enterococcus faecalis* [54] and the angiosperm *Allium sativum* [55].

The TEM results of SeNPs synthesized from the *Desmonostoc alborizicum* cyanobacterial extract showed that the nanoparticles were spherical and agglomerated. When more precision and magnification are used, the rod agglomerates of SeNPs formed are actually a collection of spherical particles which make the surface uneven and it is possible that SeNPs are held together by van der Waals forces [56]. Similar to the results obtained in this research, Afzal et al. [42] during an investigation concerning the synthesis of SeNPs from an extract of the cyanobacterium *Anabaena variabilis* showed that the SeNPs synthesized by the green method had a spherical shape. Furthermore, Zinicovscaia et al. [57] also showed that the SeNPs synthesized by the green method with the help of *Nostoc biomass* were spherical in shape. During investigations of the green synthesis of SeNPs and their morphological properties, TEM images of the arranged SeNPs exhibited a unified circulation and proved their ball-shaped dimorphism [58]. The ball-like construction of SeNPs in TEM images was between 3 and 18 nm in diameter. They showed that a thin polymer layer enclosed the nanoballs. During the green synthesis of SeNPs and morphological examination with TEM exhibited that the synthesized SeNPs had a size between 20 and 60 nm and were spherical in shape [59].

FTIR data were analyzed based on previous studies [60] [61] [62] [63]. In the FTIR spectrum of the examined sample, the peak at the wave number 3563 cm^− 1^ is related to the stretching vibration of O-H and N-H bonds in the hydroxyl structures and amine groups in the structure of the cyanobacterial extract. Also, the peaks located at the wave number 3046 cm^− 1^ and 2946 cm^− 1^ respectively corresponded to the stretching vibration of C-H bonds in aromatic and aliphatic structures, and the peak located at the wave number 1709 cm^− 1^ is also related to the stretching vibration of the bond C = O carbonyl group in the structure of this material. Furthermore, the peaks located at the wave number of 1548 cm^− 1^ and 1413 cm^− 1^ were also related to the bending vibration of N-H bonds in the amine structure and the bending vibration of the C-H bond in the structure of this extract. Also, the peaks located at 1280 cm^− 1^ and 1114 cm^− 1^ wave numbers corresponded to the stretching vibrations of C-N and C-O bonds, respectively, and the peak located at 830 cm^− 1^ corresponded to the deformation vibration of C-H bonds. The peak located at 617 cm^− 1^ is related to the bending vibration of the aromatic ring in the structure of this extract. Also, the sharp and intense peak located at the wave number 404 cm^− 1^ is related to the SeNPs synthesized in this research.

Using X’Pert HighScore Plus Software, the XRD data was used to identify the phase of these samples based on previous studies [64] [65]. Examining the XRD results showed the hexagonal structure of selenium in this sample with only one crystalline phase identified, equivalent to the structure of selenium with reference code JCPDS No. 00-006-0362, a trigonal crystal structure.

DLS results showed the uniformity of droplet size distribution in all samples and the value of the Polydispersity Index (PDI) was reported as 0.635. The PDI indicates the homogeneity of the droplet size of the nanoparticles, the higher the dispersion value, the less the uniformity of the droplet size of the nanoparticles. Furthermore, a PDI value of 0.7 or higher is unsuitable for PDI analysis indicating samples with a very wide size distribution [66].

The frequency of particles in order of size was 0.2% (32.67 nm), 1.2% (37.84 nm), 3.2% (43.82 nm), 8.2% (50.75 nm), 84.5% (58.77 nm), 2.2% (68.06 nm) and 0.5% (78.82 nm). The obtained results were completely consistent with the results of the dimensions of nanoparticles obtained from the XRD results. The obtained results can be inaccurate for the case of nanoparticles of a completely uniform shape, because DLS does not allow the distinction between nanoparticles with slight differences in diameter, nor does it accurately represent multipart samples. This is because the DLS method measures the intensity of the scattered light, which is proportional to six times the diameter of the particle. In a multi-part sample, the scattered light from larger particles or agglomeration is strongly superimposed on the light caused by smaller particles. Compared to other analytical methods, DLS measures the hydrodynamic diameter of particles that contain hydration layers, polymer shells, or other possible entities such as stabilizers, which leads to much larger nanoparticles. This is why the measurement of hydrodynamic size obtained through DLS is obtained not only on the metal core but also with all the biological molecules present in the liquid sample [59].

TEM and DLS analysis of the formation of SeNPs synthesized from *Rosmarinus officinalis* extract, showed that the synthesized SeNPs are mostly spherical with a size of about 20 to 40 nm [67].

Similarly, analysis of TEM results and DLS analysis confirmed the formation of SeNPs synthesized from Korean isotropic *Aspergillus oryzae* extract, which were polydisperse with an average particle size of 55 nm [68], and using the green method, rod shaped SeNPs with DLS of 79.2 nm were reported [69]. The increased particle volume according to DLS detection was thought to be due to measuring the hydrodynamic measurement instead of the physical size, and using the green method synthesized SeNPs had a zeta diameter of 8.12 nm and a PDI of 0.212 [58]. Other investigations concerning the green synthesis of SeNPs from *Staphylococcus aureus* bacteria extract, with TEM results and DLS analysis, showed that the synthesized nanoparticles were spherical and had a diameter of approximately 40 to 60 nm [70].

The zeta potential of SeNPs produced in this research was equal to 22.7 mV and particles had electrophoretic mobility equal to 0.117 Vs/cm^2^ which showed good stability of produced nanoparticles. According to studies, the value of zeta potential is a very important parameter to evaluate the stability of nanoparticle suspensions. When nanoparticles are stabilized with low molecular weight surfactants, mainly through electrostatic interactions, zeta potential values above 25 mV are considered to indicate very good stability. However, when stabilization is achieved with high molecular weight stabilizers, zeta potential values of only 20 mV or less can provide sufficient repulsive forces and good stabilization [71]. SeNPs synthesized by a chemical reduction method with three different coatings of SeNPs-BSA, SeNPs-Chit and SeNPs-Gluc reported zeta potentials of + 0.27, 24 and − 45 mV, respectively [67]. The antimicrobial activity of chemically and biologically manufactured SeNPs has been assessed previously but they have various particle sizes and are prepared using different methods. However, biogenic SeNPs exhibited higher antimicrobial activity in comparison to chemically synthesized SeNPs [72]. For example, SeNPs exhibited two times more inhibition zone diameters (IZD) against *Staphylococcus aureus* in comparison to silver nanoparticles (7 and 3 mm, respectively) [73]. It was noted that the antimicrobial impact of SNPs is linked to species size. Nanoparticles are small and this leads to an increase in the surface-to-volume ratio, upgrading the biological responsiveness of the nanoparticles. These findings indicate a likely process of antibacterial activity of SeNPs which entails the production of reactive oxygen species (ROS), nanoparticle penetration in the cell, and disrupting cell survival routes [74] .

Nanomaterial-induced ROS are an essential key in apoptosis and cellular toxicity. Nonetheless the weak cytotoxic impact of biogenic SeNPs was indicated. ROS generation promotes membrane lipid peroxidation and oxidative DNA impairment and increases the leakage of the cytoplasmic content and damage to the cellular wall.

Estevez et al., 2020 [75] found that SeNPs are able to inhibit the growth of mycobacteria by damaging their cell envelope integrity. A number of studies have shown that proteins might attach to the SeNPs surface, either by means of free cysteine or amine groups within the proteins, allowing stabilization.

 [76, 77]. Similar studies have indicated that bioactive compounds, such as phenolic compounds, may bind to the surface of metallic nanoparticles [78].

In general, the results of this research showed that the use of SeNPs at a concentration of 10 µg/ml significantly increased the diameter of the absence of growth halo (increased growth inhibition) of the investigated fungi. Among the investigated fungi, *Pythium ultimum* was the most resistant to nanoparticles exposed to Se, while *Alternaria alternata* showed the highest sensitivity. Although the antifungal effect of the SeNPs was not as effective as for the positive control (nystatin), they may still be useful for controlling fungi, either through further research or in combination with other antifungal agents.

In line with the results of this research, the antifungal properties of SeNPs synthesized by the green method at concentrations of 0, 0.25, 0.5, 1 and 1.7 mg were investigated [59]. They showed that SeNPs were effective against two fungi, *Fusarium oxysporum* and *Colletotrichum gloeosporioides*. During the synthesis of SeNPs from *Trichoderma atroviride* extract, the use of nanoparticles showed a good inhibitory effect against the fungus *Pyricularia grisea* at a concentration of 100 ppm [79]. Furthermore, SeNPs synthesized by the green method showed good antifungal properties against *C. glabrata*, *Candida albicans*, *Candida krusei*, *Candida parapsilosis* and *Cryptococcus neoformans*, and this antifungal property was dose dependent [80]. Different concentrations of SeNPs (50 to 150 micrograms/ml) synthesized through green and chemical methods were tested against *F. graminearum*, *F. poae*, *F. avenaceum* and *F. culmorum* [81]. They showed that SeNPs synthesized by the green method were more effective against the tested fungi than nanoparticles synthesized by the chemical method. Similarly, a lower minimum inhibitory concentration was reported for nanoparticles synthesized by the green method. The antifungal activity of nanoparticles synthesized under in vivo conditions were reported using red pepper and tomato leaves inoculated with ***Phytophthora capsici*** and *Alternaria solani*, showing that SeNPs inhibited mycelium growth at concentrations of 50 and 100 µg/ml [79].

Salem et al. [82] suggested that nano-Se (synthesized by the green method), and nanocomposite agents with fungicidal effects were effective at controlling *P. digitatum* strains using concentrations of 10–100 µg/ml. SeNPs produced by the green method have been reported to have a wide range of antimicrobial activity against fungal pathogens in food such as *Aspergillus brasiliensis*, *A. flavus*, *A. oryzae*, *A. ochraceus*, *F. anthophilum*, and *Rhizopus stolonifera* [83], and that the use of very low concentrations of SeNPs synthesized by the green method (100 and 200 ppm) inhibited the growth of *Pyricularia grisea* [68].

The antifungal activity of SeNPs can be interpreted as a result of nanoparticles with a smaller size having higher stability and a higher surface-to-volume ratio, which leads to their antimicrobial properties that are found in the same material on a larger scale in bulk form [84]. Due to the two characteristics of nanoparticles, i.e. smaller size and high surface-to-volume ratio, they have the possibility to interact closely with the cell membrane of microorganisms, which leads to the facilitation of interactions and intracellular diffusion, and also causes significant damage to the membranes and toxic effects on DNA or leads to the inhibition of cell proliferation by reactive oxygen species-mediated processes [85]. In addition, it has been suggested that the antimicrobial activity of nanoparticles may be due to the release of ions in the environment where microorganisms grow. It is also known that the dissolution of ions depends on the size of nanoparticles and the concentration of the solution [86]. Research shows that SeNPs have size- and concentration-dependent antimicrobial effects against various microorganisms [87].

## Conclusion

In this study, the synthesis of SeNPs was done by the green method using an extract from the cyanobacterium *Desmonostoc alborizicum*. UV spectroscopy of nanoparticles showed the formation of a peak in the range of 271–275 nm and the presence of only one SPR band in the absorption spectrum that indicated the synthesis of spherical nanoparticles. The spherical nature of the nanoparticles was consistent with the results obtained from the TEM test, and the formation of spherical nanoparticles and the agglomerated state were confirmed. FTIR and XRD tests confirmed the formation of new bonds based on the synthesis of SeNPs using the *Desmonostoc alborizicum* extract. The investigation of dispersion distribution of synthesized nanoparticles showed the PDI value was equal to 0.635, which indicated the homogeneity of droplet size in nanoparticles. According to the results of DLS, the frequencies of particle sizes were 0.2% (32.67 nm), 1.2% (37.84 nm), 3.2% (43.82 nm), 8.2% (75 50.50 nm), 84.5% (58.77 nm), 2.2% (68.06 nm) and 0.5% (78.82 nm). The zeta potential of the produced SeNPs was 22.7 mV and the electrophoretic mobility of the particles was 0.117 cm^2^/Vs, which indicated the stability of the synthesized SeNPs. Antifungal properties of prepared SeNPs against fungal strains investigated in this research, which increased in a concentration-dependent manner, showed that the most sensitive among the strains was related to *Alternaria alternata* and the highest resistance was reported in *Pythium ultimum*.

Undoubtedly, our understanding in the field of cyanobacterial nanoparticles has significantly improved in the past decade, but there are still many challenges to their application for their nanoformulation and commercialization. Many natural compound-based drugs against fungae have been discovered, but they have poor water solubility, bioavailability, instability, and insolubility, which makes their formulation difficult or even impossible. Cost effective production processes with natural cyanobacterial compounds may cause major limitations for commercialized platforms. Therefore, researchers are moving to a diversified field of nanoformulations for cost effective and rapid developments.

Uncovering novel enhanced anti-fungal mechanisms of nanoformulations consisting of cyanobacterial natural products will add to the development of nano-drugs. These findings have increased the interest in nanoparticles biosynthesized by cyanobacteria and drugs from cyanobacteria as an alternative to expensive antifungal drugs. By developing bioactive cyanobacterial nanoformulation drugs, it will be possible to build data sets for cyanobacteria derived pharmaceuticals. However, further research should be performed to achieve innovation in commercialized platforms. Scale-up commercialization with technology development should also be a major task for cost effective cyanobacterial nanoformulations. Therefore, future works should explore the novel functions of cyanobacterial natural products.

### Electronic supplementary material

Below is the link to the electronic supplementary material.

Supplementary Material 1

Supplementary Material 2

Supplementary Material 3

Supplementary Material 4

## Data Availability

All data generated or analysed during this study are included in this published article (supplementary information files: S1, S2 and S3).

## Abbreviations

SeNPs: Selenium nanoparticles
PDI: Polydispersity index
*mcy*: Genes of the microcystin synthetase
CCC: Cyanobacteria Culture Collection
TEM: Transmission electron microscope
XRD: X-Ray Diffraction,
DLS: Dynamic Light Scattering
ROS: Reactive oxygen species
Se: Selenium
SDA: Sabouraud dextrose agar
PVDF: Polyvinylidene fluoride
FTIR: Fourier-transform infrared spectroscopy
IROST: Organization for Science and Technology

## Declarations

### Live vertebrates and/or higher invertebrates

This study is not on live vertebrates and/or higher invertebrates.

### Images

All the images are original and not copy from other papers.
