# Supplementary material for: Antifungal screening of selenium nanoparticles biosynthesized by microcystin-producing Desmonostoc alborizicum
Source: BMC Biotechnol. 2023 Sep 27;23:41. doi: 10.1186/s12896-023-00807-4 (PMC10538242; doi:10.1186/s12896-023-00807-4)
Supplement: Supplementary file 1 — Supplementary Material 1 [file 12896_2023_807_MOESM1_ESM.docx]

**Sub figure legend:**

**Supplementary Fig. S1:** Raw data related to FT-IR

**Supplementary Fig.** **S2:** Raw data related to XRD

**Supplementary Fig. S3:** Raw data for Antifungal Activity of Silver Nanoparticles
